# Hydroxychloride trace elements improved eggshell quality partly by modulating uterus histological structure and inflammatory cytokines expression in aged laying hens

**DOI:** 10.1016/j.psj.2021.101453

**Published:** 2021-08-28

**Authors:** Qiuyu Jiang, Jingjing Sun, Yang He, Yanbo Ma, Bingkun Zhang, Yanming Han, Yuanyuan Wu

**Affiliations:** ⁎ State Key Laboratory of Animal Nutrition, China Agricultural University, Beijing 100193, China; † Department of Animal Physiology, College of Animal Science and Technology, Henan University of Science and Technology, Luoyang 471023, China; ‡ Trouw Nutrition R&D, Amersfoort, the Netherlands

**Keywords:** hydroxychloride, eggshell ultrastructure, inflammatory cytokines, uterus, laying hen

## Abstract

The objectives of this study were to investigate the effectiveness of dietary zinc, copper, and manganese hydroxychloride (**HC**) supplementation on performance, minerals deposition, serum parameters, eggshell ultrastructure, uterus histological structure, and inflammatory cytokines in aged hens. A total of 560 Hyline Brown layers at 62 wk of age were randomly allotted into 3 groups (CON, basal diet without extra minerals supplemented; Sulphate and HC, basal diet with sulphate or hydroxychloride zinc, copper, and manganese supplementation at levels of 80, 15, and 80 mg/kg, respectively). The trial lasted for 16 wk consisting of 4 wk depletion period and 12 wk testing period. The results indicated that dietary hydroxychloride trace elements increased egg weight (*P* < 0.05) when compared with CON group and improved average Haugh unit and albumen height (*P* < 0.05) when compared with Sulphate group from 70 to 73 wk. Trace element supplementation significantly increased eggshell strength, ceruloplasmin content in serum, and modified crystallographic structure of eggshell (*P* < 0.05) that included effective layer height, palisade height, mammillary layer width, and mammillary internal area ratio, but the results did not differ regarding the trace mineral sources used. Furthermore, hens fed with hydroxychloride trace element showed the highest mucosal fold height (*P* < 0.05) and epithelial height (*P* = 0.053) in eggshell gland, as well as mRNA expression of *TNF*-*α* (*P* < 0.05) and *IL*-*22* (*P* = 0.094). It is concluded that supplementation of Zn, Cu, and Mn mixture modified eggshell quality partly through enhancing histological structure and immune responses of uterus. Hydroxychloride source of Zn, Cu, and Mn excelled sulphate in its beneficial effects for birds.

## INTRODUCTION

The problem of lower egg shell quality caused by prolonged egg production period is an important issue affecting the breeding of old layer hens. Approximately 10% of the eggs produced in poultry farms are lost due to breakage of eggshells, which accounts for huge economic loss to the egg industry (Sah et al., 2018). Improving eggshell quality are essential for protection against penetrating of pathogenic bacteria (Swiatkiewicz and Koreleski, 2008) and reducing egg damage thus enhancing economic efficiency. Eggshell quality also reflects reproductive condition where eggshell formed. Prolonging the egg production cycle destroys the integrity of the endometrium, resulting in eggshell microstructure defects and eggshell quality degradation. Eggshell microstructure consists of shell membrane, mammillary layer, palisade layer, crystal surface layer, and cuticle from inside to outside (Hincke et al., 2012), whereas the palisade layer, crystal surface layer, and cuticle constitute effective layer. Relevant studies show that the height of effective layer, palisade layer, and the density of mammillary knobs affect the strength of the eggshell (Qiu et al., 2020).

A great deal of efforts have been applied to improving eggshell quality in old laying hens in the fields of mineral nutrition (Xiao et al., 2015; Esfahani et al., 2020), which involved in the synthesis of calcium crystals during shell formation as a function of catalytic enzymes. Zn, Cu and Mn supplementation improved the eggshell structural characteristics with higher palisade layer thickness (Stefanello et al., 2014). On the contrary, the eggshells of minerals deficient hens are characterized by an abnormal distribution of the shell membranes (Mabe et al., 2003) and alterations in mammillary layer (Leach and Gross, 1983), which resulted in egg shape deformation and poor mechanical properties. To avoid these problems, inorganic trace minerals like oxides, sulfates, carbonates, and phosphates are supplemented at higher concentrations in diet, which was reported to be unfriendly to the environment and even detrimental to performance of birds (Brugger and Windisch, 2015). For instance, oviductal tissue regression and regeneration in laying hens that induced by high level of zinc in diet was identified by Jeong et al. (2013). The trace elements in hydroxychloride form are expected because it has shown reduced oxidation in feed during storage due to its low solubility in water (Cao et al., 2000; Mavromichalis et al., 2000).In addition, hydroxychloride trace elements prevent from binding with dietary constituents and forming of indigestible complexes (Huang et al., 2013).

Numerous researchers have focused on the addition of trace elements in the diet to regulate egg quality by improving eggshell ultrastructure (Fathi et al., 2016). The mechanism of oviduct especially the uterus, in which the eggshell formed, is still unclear when working with minerals. The poultry uterus or shell gland is highly complex and dynamic that substantial in the mineralization of the eggshell, especially in the growth of the crystal. Calbindin 1, ovocleidin-116, and phosphoprotein 1 secreted from the shell gland have an active role on CaCO_3_ growth, aggregation and inhibition (Hernández et al., 2008), contributing in remodeling for eggshell mineralization. Other proteins secreted by the uterus participate in the correct folding of the eggshell matrix or possess antibacterial properties, which can protect the eggs from microbial invasion (Jonchere et al., 2010). Damaged endometrial tissue that associated with increasing hen age inhibit the processes of ion transmission and the crystallization of eggshell formation, resulting in a large and nonuniform mammillary knob formation (Park and Sohn, 2018). Furthermore, the inflammatory cytokines in the chicken oviduct may play roles not only in the immune response that against bacterial infection, but also in the process of eggshell mineralization (Elhamouly et al., 2018). It is clear that morphological changes and weakened immune response in the oviduct caused by aging will adversely affect the egg laying process, as well as quality of eggs. The data concerning minerals Zn, Cu, and Mn on uterus histological structure and immune parameters of layers are scant, and the previous findings about the effects of trace element on eggshell quality of laying hens resulted in contradictory conclusions. Therefore, the objective of the current study aimed to providing a baseline of the relationship among the microminerals, uterus status, and eggshell ultrastructure by evaluating the impacts of hydroxychloride Zn, Cu, and Mn mixture on performance, serum parameters, eggshell ultrastructure, uterus histological structure, and inflammatory cytokines of aged hens.

## MATERIALS AND METHODS

### Birds, Diets, and Experimental Design

The experiment was conducted at ZhuoZhou poultry Research Unit at Hebei province, China, and approved by Animal Care and Use committee of China Agriculture University. A total of 560 sixty two-wk-old Hyline Brown layers with similar body weight and performance were randomly assigned to 3 treatments. The experimental treatments were as follows: 1) CON group (basal diet without extra Zn, Cu, and Mn supplemented); 2) Sulphates group (basal diet + 80ppm Zn, 15ppm Cu, and 80ppm Mn from sulphate; 3) HC group (basal diet + 80 ppm Zn, 15 ppm Cu, and 80 ppm Mn from hydroxychloride). The control group had 8 replicates with 10 birds per replicate, the other 2 groups each had 15 replicates with 16 birds per replicate. Two hens were allocated to one battery cage (40 cm × 37 cm × 34 cm) that equipped with nipple drinker under a 16L:8D lighting regimen. Average minimum and maximum temperature, as monitored inside the shed on a daily basis, were 23°C and 26°C, respectively, and the RH average values recorded were 37.6% (minimum) and 74% (maximum).

The trial lasted for 16 wk, including 4 wk of depletion period and 12 wk of testing period. During the depletion period, the same basal diet was provided for the birds and no extra Zn, Cu, and Mn has been supplied. Basal diet with the typical commercial ingredients was prepared for the formal experiment, and the dietary components and nutrient levels were presented in Table 1. The content of Zn, Cu, and Mn in diet (especially limestone, which was replaced by medical stone) was strictly controlled. The hydroxychloride minerals were provided as IntelliBond Zn, Cu, and Mn by Trouw Nutrition. The sulphates minerals were also supplied by Trouw Nutrition. IntelliBond minerals deliver 36, 25, and 32% Zn, Cu, and Mn, and sulphates minerals deliver 58, 58, and 44% Zn, Cu, and Mn.

**Table 1:** Basal diet composition and nutrient level.

Ingredients	Content (%)	Nutrient levels 2	Content (%)
Corn	41.2	Metabolic energy (kcal/kg)	2,690
Soybean	13.0	Crude protein	14.0
Wheat	34.0	Calcium	3.78
CaCO_3_	9.60	Available phosphorus	0.30
CaHPO_4_	1.00	Lysine	0.68
L-Met	0.09	Methionine	0.32
Lys	0.15	Methionine + Cystine	0.56
Choline chloride	0.10		
NaCl	0.30		
Premix^1^	0.20		
Antioxidant	0.02		
Medical stone	0.34		
Total	100		

1 Provided the following per kg of diets: Fe, 80 mg (FeSO_4_); Se, 0.25 mg (Na_2_SeO_3_); I, 2.2 mg (Ca(IO_3_)_2_); Vitamin A, 9,000 IU (retinylacetate); Vitamin D_3_, 30,00 IU; Vitamin K_3_, 2.65 mg; Vitamin B_1_, 2 mg; Vitamin B_2_, 10 mg; Vitamin B_12_, 0.025 mg; Vitamin E, 35 IU (α-tocopherol acetate); Biotin, 0.25 mg; Folic acid, 1.5 mg; Pantothenic acid, 12 mg; Niacin, 50 mg. No Cu, Mn, and Zn were supplemented.

2 Nutrient levels were calculated values.

### Sample Collection

At the end of the experimental period, based on the records of laying time and oviposition cycle, at 16 h after laying hens, approximately 3 mL of blood from each bird per replicate was drawn from brachial vein into a centrifuge tube. One egg per replicate was randomly collected; the eggshell was taken for scanning electron microscopy (**SEM**). One bird per replicate were randomly selected and slaughtered. The reproductive tract was dissected out from the thoracoabdominal cavity immediately. Approximately 1 cm^2^ of uterus were cut from the same position and fixed into 4% neutral paraformaldehyde solution for having an assessment of histological structure in oviduct, another collected uterus sample was rinsed with physiological saline and stored at −80°C until mRNA expression analysis.

### Laying Performance

Egg number, egg weight and broken egg number were recorded daily, while feed intake was registered weekly. The feed conversion ratio (**FCR**, g:g) was calculated from egg production, egg weight, and feed intake per 4 wk.

### Serum Parameters Related to Minerals

Serum was apportioned by centrifuging the blood for 10 min at 3,000 rpm and then preserved at −80°C for measuring the degree of ceruloplasmin (**CP**), total calcium (**Ca**), super oxidase dismutase (**SOD**), alkaline phosphatase (**ALP**), and carbonic anhydrases (**CA**). An automatic biochemical analyzer (iChem 340, Shenzhen Icubio Biomedical Technology Co., Ltd., Shenzhen, China) was used to determine parameters related with minerals in serum according to operating standards, except for CA that was analyzed using enzyme-linked immunosorbent assay (Shanghai Ji Chun industrial Co., Ltd., Shanghai, China).

### Egg Quality and Eggshell Ultrastructure

All collected eggs on the last day of 67, 71, and 77 wk were used to assess the egg quality parameters. Eggshell strength, albumin height, and Haugh unit were measured with a digital egg tester (EMT-5200, Robotmation Co., Ltd., Tokyo, Japan), and eggshell thickness was determined by measuring the mean values taken at 3 locations on the egg by using a thickness Gauge (ESTG-1, ORKA Food Technology Ltd., Ramat Hasharon, Israel). After measuring parameters, broke egg and weigh egg yolk.

The collected eggs at the end of the experiment were broken from the sharp end and removed contents, boiled in 5% NaOH solution for 10 min to remove the eggshell membranes, and then dried. One piece of eggshell approximately 1 cm^2^ from the equatorial section of each sampled egg was examined by scanning electronic microscopy (SU8020, HITACHI., Tokyo, Japan). The effective layer height, mammillary layer height, and the mammillae width were measured 6 replicates per slice under magnification condition of 200 times and calculated with the image pro plus 6.0 according to the model by Hincke et al. (2012). The effective layer height was measured by drawing a line from top of palisade layer to cuticle. Under 100 times magnification, the inner surface of the eggshell was photographed and stored by SEM for measuring the mammillary knob density and mammillary internal area. The mammillary parameters were calculated as follows: mammillary internal area ratio = mammillary internal area/mineralized area; mammillary density = the number of mammillae/square millimeter.

### Uterus Histological Structure, mRNA Expression of Inflammatory Cytokines

After staining with hematoxylin and eosin (**HE**), sections of the uterine samples were examined under optical microscope (DM750, Leica, Frankfurt, Germany) at a magnification 100 × and 400 × for morphometry analysis including the mucosal fold height, epithelial height, glandular width, and glandular luminal diameters. The height of mucosal fold was measured by drawing a vertical line from the epithelial to the fold. Epithelial height was determined by measuring the height of 15 cells in 5 different primary folds (Berg et al., 2001).The individual glandular width was measured by drawing a perpendicular line across the widest part of the gland. Glandular luminal diameter was determined by measuring perpendicular distance between 2 opposing gland cells (Kimaro et al., 2013). The mucosal fold height, glandular width and glandular luminal diameter were measured 6 replicates per tissue slice. The uterine measurements were made using image pro plus 6.0 processing and analysis software.

Total RNA was isolated from uterus mucosa using Trizol Reagent kit according to the manufacture's instruction. A260 and A280 measurements were used to spectrophotometrically determine the RNA concentration and purity. Reverse transcription (**RT**) procedure was 37°C, 5 min, followed by 42°C, 15 min, and then 85°C, 5 min. The products cDNA was stored at −20°C until PCR measurement. A total volume of 20 µL PCR reaction system that contained 10 µL PCR Master Mix, and 0.8 µL primer (0.4 µL forward and 0.4 L reverse), and 2 µL cDNA template, and 7.2 µL sterile superstilled water was used. The primers are represented in Table 2. Gene expression contents were assessed by a real time quantitative PCR using Thermo fisher 7500 and the following profiles: denature at 95°C for 15 s, 40 cycles at 95°C for 15 s, 60°C for 1 min. At the end of the PCR reactions, a melt curve analysis was performed for all genes. The Ct value was determined and used to calculate relative expression level 2^−ΔΔCT^.

**Table 2:** Sequences of the primers used in real-time PCR assays.

Gene name^1^	Gene bank ID	Primer sequence(5’-3’)^2^	Fragment size (bp)
*IL-1β*	NM_204524.1	F:TGGGCATCAAGGGCTACA R:TCGGGTTGGTTGGTGATG	244
*IL-6*	NM_204628.1	F:CAAGGTGACGGAGGAGGAC R:TGGCGAGGAGGGATTTCT	254
*IL-22*	NM_001199614.1	F:GAATCACAGCAAAGCGCTGA R:TGTAGGGCTGCTGGAAGTTG	230
*TNF-α*	NM_204267.1	F:GAGCGTTGACTTGGCTGTC R:AAGCAACAACCAGCTATGCAC	64
*CATH1*	NM_001001605.3	F:TCAGCTGCTCCTCACCAGTC R:ATTGGCCCTGAAGTTGGGCA	223
*CaBpD28k*	NM_205513	F:TTAAATCTGCGTTGCTTCCATACA	298
		R:GGCCCATCCTGCACTCCATAAC	
*Β-actin*	NM_205518.1	F:CCACCGCAAATGCTTCTAAAC R:AAGACTGCTGCTGACACCTTC	175

1 *IL-1β*, interleukin-1β; *IL-6*, interleukin-6; *IL-22*, interleukin-22; *TNF-α*, tumor necrosis factor α;*CATH1*, cathelicidin 1.

2 F, forward; R, reverse.

### Statistical Analysis

All analysis was performed with SPSS 24.0 software. The data were analyzed by general linear model. Differences between treatments were detected by Duncan's multiple-range test and significant differences were considered at *P* < 0.05. The results were expressed as mean and SEM.

## RESULTS

Table 3 showed the supplemental mineral contents of the diets together with levels of assayed and calculated mineral contents. The assayed mineral content was close to the calculated content.

**Table 3:** Concentrations of minerals in experimental treatment diets (air-dry basis).^1^

Group^2^	Zn (ppm)			Cu (ppm)			Mn (ppm)		
	Supplemented	Calculated	Assayed	Supplemented	Calculated	Assayed	Supplemented	Calculated	Assayed
CON^3^	0	30.22	30.22	0	6.68	6.68	0	21.38	21.38
Sulphate	80	110.22	117.83	15	21.68	20.04	80	101.38	93.35
HC	80	110.22	125.96	15	21.68	24.66	80	101.38	105.00

1 Data represent the means from 8 hens in CON group and 15 hens in sulphates and HC group.

2 CON: the basal diet without extra Zn, Cu, and Mn supplemented; Sulphate: basal diet + 15 ppm Cu, 80 ppm Zn, and 80 ppm Mn from sulphate; HC: basal diet + 15 ppm Cu, 80 ppm Zn, and 80 ppm Mn from hydroxychloride.

3 The Zn, Cu, and Mn content in the basal diet were 30.22 ppm, 6.68 ppm, and 21.38 ppm, respectively.

### Performance

The data of laying performance in aged hens from 66 to 77 wk are listed in Table 4. Egg rate did not differ (*P* > 0.05) among the treatments in the whole period, although it was numerically higher in HC group in 70 to 73 wk and 74 to 77 wk. Layers fed hydroxychloride trace element exhibited the highest egg weight (*P* < 0.05) and the lowest FCR (*P* = 0.058) in 70 to 73 wk, as well as the highest egg weight (*P* = 0.073) in 74 to 77 wk. In addition, compared with CON group, dietary supplementation with minerals irrespective of source remarkably decreased the FCR and broken egg ratio in 74 to 77 wk (*P* < 0.05).

**Table 4:** Effects of sulphate and hydroxychloride trace elements on laying performance of Hyline Brown-laying hens from 66 to 77 wk of age.^1^

Items	Zn-Cu-Mn supplementation^2^				SEM	*P*-value
	CON	Sulphate		HC		
Egg rate (%)						
66–69 wk	83.38	83.53		82.87	0.907	0.948
70–73 wk	81.50	83.73		84.20	0.917	0.547
74–77 wk	78.13	79.64		80.20	0.979	0.737
66–77 wk	80.82	82.59		82.42	0.872	0.741
Average egg weight (g)						
66–69 wk	60.95	61.59		61.44	0.209	0.529
70–73 wk	61.89^b^	62.53^ab^		63.10^a^	0.182	0.048
74–77 wk	62.53	62.78		63.46	0.216	0.073
66–77 wk	61.87	62.37		62.64	0.186	0.321
Feed conversion ratio (g:g)						
66–69 wk	2.16		2.12	2.16	0.019	0.558
70–73 wk	2.25		2.15	2.13	0.019	0.058
74–77 wk	2.36^a^		2.16^b^	2.22^b^	0.028	0.027
66–77 wk	2.25		2.14	2.17	0.019	0.087
Broken egg ratio (%)						
66–69 wk	1.32		1.07	0.84	0.129	0.385
70–73 wk	1.69		1.22	1.05	0.128	0.176
74–77 wk	2.20^a^		1.34^b^	1.40^b^	0.143	0.043
66–77 wk	1.70		1.18	1.30	0.113	0.282

1 Data represent the means from 8 replicates (10 hens per replicate) in CON group and 15 replicates (16 hens per replicate) in Sulphate and HC group.

2 CON: the basal diet without extra Zn, Cu and Mn supplemented; Sulphate: basal diet + 15 ppm Cu, 80 ppm Zn, and 80 ppm Mn from sulphate; HC: basal diet + 15 ppm Cu, 80 ppm Zn, and 80 ppm Mn from hydroxychloride.

a-b Means with different superscripts within the same row are significantly different (*P* < 0.05).

### Serum Parameters Related to Minerals

Data exhibited in Table 5 summarize the impact of dietary Zn, Cu, and Mn supplementation on serum parameters of laying hens at wk 77. Compared with CON group, bird fed with sulphate and hydroxychloride minerals showed significantly increased ceruloplasmin activity (*P* < 0.05). In addition, total calcium content (*P* < 0.05) and SOD activity (*P* > 0.05) was the highest in hydroxychloride group, but differences between Sulphate group and HC group were not significant.

**Table 5:** Effects of sulphate and hydroxychloride trace elements on serum parameters of Hyline Brown-laying hens at 77 wk of age.^1^

Items	Zn-Cu-Mn supplementation 2			SEM	*P*-value
	CON	Sulphate	HC		
Ceruloplasmin (IU/L)	129.23^b^	148.86^a^	151.57^a^	2.376	0.001
Total calcium (mmol/L)	4.45^b^	4.57^ab^	4.79^a^	0.055	0.036
Super oxidase dismutase activity (IU/L)	26.73	25.54	27.54	0.849	0.604
Alkaline phosphatase (U/L)	180.20	202.35	193.08	15.744	0.885
Carbonic anhydrases(U/L)	29.38	32.59	29.58	1.153	0.439

1 Data represent the means from 8 hens in CON group and 15 hens in Sulphate and HC group

2 CON: the basal diet without extra Zn, Cu, and Mn supplemented; Sulphate: basal diet + 15 ppm Cu, 80 ppm Zn, and 80 ppm Mn from sulphate; HC: basal diet + 15 ppm Cu, 80 ppm Zn, and 80 ppm Mn from hydroxychloride.

a-b Means with different superscripts within the same row are significantly different (*P* < 0.05).

### Egg Quality

As is shown in Table 6, addition of trace elements to the diet dramatically increased eggshell strength (*P* < 0.05), whereas no significant differences were observed between 2 different sources. Furthermore, Haugh unit and albumen height were increased (*P* < 0.05) when sulphate substituted with hydroxychloride minerals. Unexpectedly, birds in CON group exhibited slightly higher Haugh unit than those in Sulphate group.

**Table 6:** Effects of sulphate and hydroxychloride trace elements on egg quality of Hyline Brown-laying hens from 66 to 77 wk of age.^1^

Items	Zn-Cu-Mn supplementation 2			SEM		*P*-value
	CON	Sulphate	HC			
Eggshell strength (N)	29.12^b^	31.26^a^	30.83^a^	0.030	0.020	
Eggshell thickness (mm)	0.34	0.35	0.35	0.003	0.089	
Haugh unit	76.83^ab^	75.15^b^	79.17^a^	0.656	0.020	
Albumen height (mm)	6.30^b^	6.09^b^	6.70^a^	0.086	0.004	
Egg yolk weight (g)	14.71	14.96	14.86	0.040	0.059	

1 Data represent the means from 8 replicates (10 hens per replicate) in CON group and 15 replicates (16 hens per replicate) in Sulphate and HC group. Data are mean values of 67, 71, and 77 wk.

2 CON: the basal diet without extra Zn, Cu and Mn supplemented; Sulphate: basal diet + 15 ppm Cu, 80 ppm Zn, and 80 ppm Mn from sulphate; HC: basal diet + 15 ppm Cu, 80 ppm Zn, and 80 ppm Mn from hydroxychloride.

a-b Means with different superscripts within the same row are significantly different (*P* < 0.05).

### Eggshell Ultrastructure

**Figure 1:**
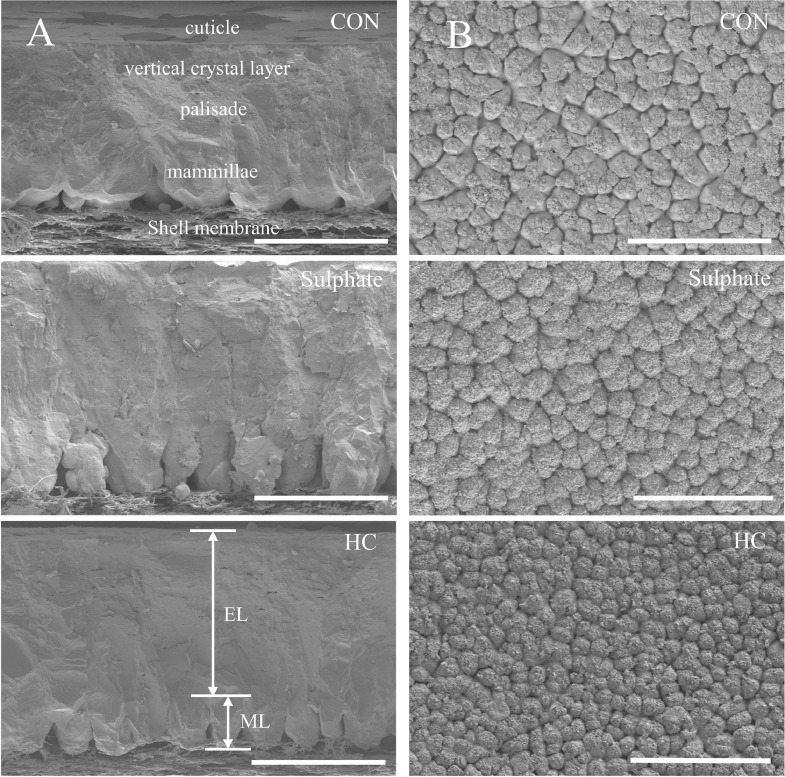
Scanning electron microscopy of eggshell cross section (A) and vertical view (B) of the mammillary knobs of laying hens at 77 wk of age. CON: the basal diet without extra Zn, Cu, and Mn supplemented; Sulphate: supplementation of 80-15-80 ppm of Zn, Cu, and Mn from sulphate source; HC: supplementation of 80-15-80 ppm of Zn, Cu and Mn from hydroxychloride source. Scale bar: A, 200 um; B, 500 um. Abbreviations: EL, effective layer; ML, mammillary layer.

Measurements of eggshell ultrastructure from cross section and vertical view are depicted at Figure 1. Compared with CON group, the inclusion of trace minerals, regardless of sulphate or hydroxychloride, did not alter (*P* > 0.05) the mammillary layer height (Table 7). However, effective layer height and palisade height were found to be significantly increased (*P* < 0.05) in Sulphate and HC groups. Adding trace elements to the diet, irrespective of sulphate or hydroxychloride sources, decreased the mammillae width and mammillary internal area ratio (*P* < 0.05), whereas mammillary knob density was not affected (*P* > 0.05) by the dietary trace elements supplementation.

**Table 7:** Effects of sulphate and hydroxychloride trace elements on eggshell ultrastructure of Hyline Brown-laying hens at 77 wk of age.^1^

Items	Zn-Cu-Mn supplementation 2				*P*-value
	CON	Sulphate	HC	SEM	
Effective layer height(um) 3	191.46^b^	224.10^a^	225.94^a^	4.615	0.008
Palisade height(um) 3	148.75^b^	177.40^a^	178.00^a^	4.305	0.017
Mammillary layer height (um) 3	107.75	109.20	115.98	2.676	0.422
Mammillae width (um) 3	87.20^a^	73.94^b^	67.27^b^	2.179	0.001
Mammillary internal area ratio (%)^4^	14.62^a^	12.02^b^	11.75^b^	0.369	0.006
Mammillary knob density (mm^2^)^5^	147.52	146.05	155.11	5.300	0.739

1 Data represent the means from 8 hens in CON group and 15 hens in Sulphate or HC groups.

2 CON: the basal diet without extra Zn, Cu, and Mn supplemented; Sulphate: basal diet + 15 ppm Cu, 80 ppm Zn, and 80 ppm Mn from sulphate; HC: basal diet + 15 ppm Cu, 80 ppm Zn, and 80 ppm Mn from hydroxychloride.

3 Data are mean values and calculated for 6 replicates per scanning electron microscope photograph.

4 Mammillary internal area ratio = mammillary internal area/mineralized area ratio.

5 Mammillary knob density = the number of mammillae/square millimeter.

a-b Means with different superscripts within the same row are significantly different (*P* < 0.05).

### Uterus Histological Structure and mRNA Expression of Inflammatory Cytokines

**Figure 2:**
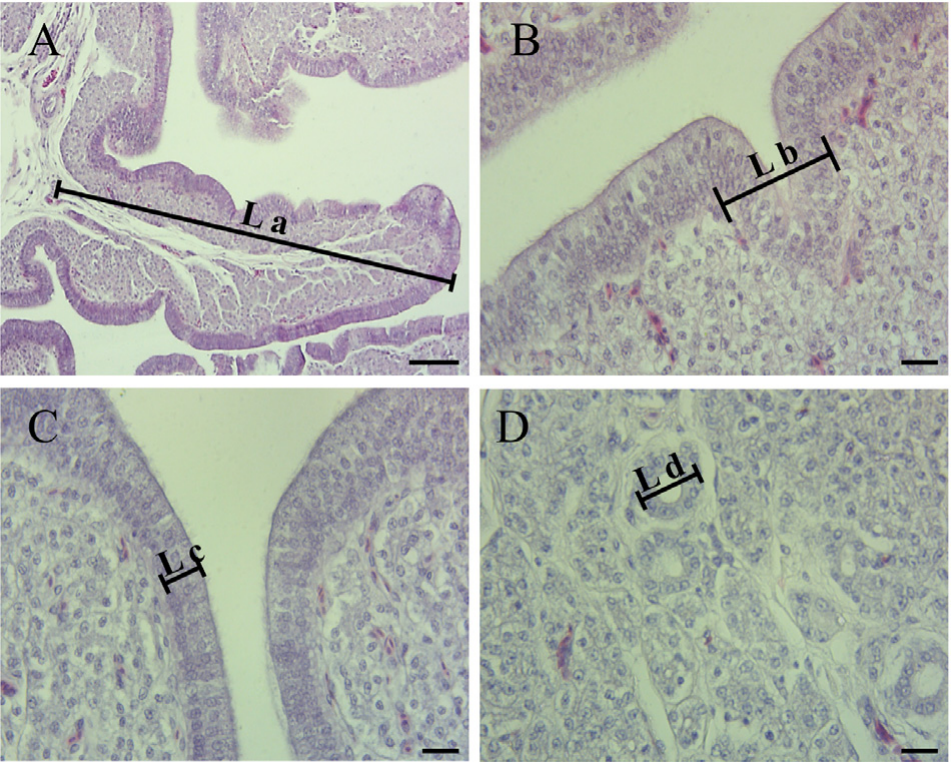
The histological structure of uterus in laying hens at 77 wk of age. (A) Low magnification showing the muscosal fold. The height of muscosal fold was indicated by La. A higher magnification photomicrograph of the height of gland, epithelium, and the diameter of the glandular luminal were shown by (B), (C), and (D). Scale bar: A, 100 um; B, C and D, 20 um.

The histological structure of uterus was depicted in the Figure 2. As is shown in Table 8, diets supplemented with hydroxychloride minerals increased (*P* < 0.05) mucosal fold height and had a tendency to improve epithelial height (*P* = 0.053) when compared with Sulphate group. In addition, mRNA expression of tumor necrosis factor α (***TNF-α***) in eggshell gland was statistically improved (*P* < 0.05) following dietary hydroxychloride trace element supplementation, whereas no significant differences between sulphate and hydroxychloride were observed. Moreover, dietary supplementation of minerals numerically increased interleukin 22 (***IL-22***) (*P* = 0.094) expression in uterus.

**Table 8:** Effects of sulphate and hydroxychloride trace elements on inflammatory cytokines expression in eggshell gland and uterus histological structure of Hyline Brown-laying hens at 77 wk of age.^1^

Items	Zn-Cu-Mn supplementation 2				SEM	*P*-value
	CON	Sulphate		HC		
CaBpD28k	1.00		0.31	0.542	0.162	0.316
Inflammatory cytokines						
*IL-1β*	1.00		1.57	1.67	0.260	0.628
*IL-6*	1.00		1.18	1.11	0.096	0.789
*IL-22*	1.00		2.03	2.57	0.268	0.094
*TNF-α*	1.00^b^		2.01^ab^	4.08^a^	0.507	0.047
*CATH1*	1.00		1.06	1.74	0.234	0.350
Uterus histological structure						
Muscosal fold height (um)	505.97^b^		506.1^b^	576.3^a^	13.593	0.039
Glandular width (um)	63.07		67.38	70.25	1.093	0.512
Glandular luminal diameters (um)	32.67		27.02	30.65	0.993	0.082
Epithelial height(um)^3^	26.32		26.24	28.66	0.500	0.053

1 Data represent the means from 8 hens in CON group and 15 hens in Sulphate or HC groups.

2 CON: the basal diet without extra Zn, Cu, and Mn supplemented; Sulphate: basal diet + 15 ppm Cu, 80 ppm Zn, and 80 ppm Mn from sulphate; HC: basal diet + 15 ppm Cu, 80 ppm Zn, and 80 ppm Mn from hydroxychloride.

3 Data are mean values and calculated the height of 15 cells in 5 different primary folds.

a-b Means with different superscripts within the same row are significantly different (*P* < 0.05).

## DISCUSSION

The study indicated that dietary Zn-Cu-Mn supplementation did not affect egg rate, which was consistent with Xiao et al. (2015). The superiority of the hydroxychloride trace elements in FCR, egg weight, as well as Haugh unit and albumen height showed up at the later phase (70–77 wk). The positive effect of Zn-Cu-Mn mixture on egg weight, Haugh unit and albumen height may be due to its role in protein synthesis and deposition of albumen in the magnum (Farhadi Javid et al., 2021). Zn, Cu and Mn were considered as cofactor of enzymes that attributed to DNA, protein and carbohydrate metabolic processes. Moreover, Abd et al. (2018) postulated that the improved egg weight may be returned to the vital role of minerals in division and multiplication of cell. Partly agreed to our study, Saleh et al. (2020) illustrated that the inclusion of 1 g/kg organic Zn, Cu, and Mn significantly improved egg production, egg weight, and FCR. Contrary to our study, the supplementation of Zn did not affect egg weight, which was found by Cufadar et al. (2020). Combination of sulphate minerals tended to decrease the Haugh unit from 66 to 77 wk compared with control group, which was unexpected. The mechanism for this was probably because of the antagonism occurring between Zn and other minerals (Min et al., 2018a; Gajula et al., 2011) when the sulphate form, but not hydroxychloride included in the diet. Similarly, the interference between minerals was observed in egg production as reported by Lim and Paik (2003).

Compared with CON group, the inclusion of minerals decreased broken egg ratio in the late phase of the trial. More consistently, aged hens of 77 wk fed with minerals, irrespective of sulphate and hydroxychloride, showed remarkable increase in eggshell strength that supported by several studies (Stefanello et al., 2014; Swiatkiewicz and Koreleski, 2008). However, dietary minerals supplementation did not alter eggshell thickness, which was inconsistent with those obtained by Saleh et al. (2020). Many studies (Qiu et al., 2020) revealed that simply measuring the eggshell thickness did not give a comprehensive understanding of the mechanical properties of eggshell, so it was necessary to evaluate eggshell ultrastructure when working with trace minerals. Eggs with an abnormal appearance may be rejected by the consumer and defects in eggshell ultrastructure can result in breakage. Furthermore, poor quality eggshells with low resistance tend to have larger mammillary knobs and higher internal area or irregular arrangement (Li et al., 2018; Park and Sohn, 2018). Zn, Cu, and Mn are essential for carbonate and muco-polysaccharides synthesis, which plays an important role in eggshell formation (Zhang et al., 2017a). Zhang et al. (2018) found that dietary Mn supplementation participated in the metabolism and genes expression of proteoglycans and glycoproteins in the eggshell gland, thus increasing the mammillary knob density during the initial phase of shell formation. In our study, the increased effective layer height, palisade layer height and decreased mammillae width and mammillary internal area ratio were found in sulphate and HC group. These positive changes played important roles in breaking strength thus contributed to alleviating the negative effect of aging on the egg quality (Qiu et al., 2020). Furthermore, our study suggests that the addition of sulphate and HC combined Zn-Cu-Mn mixture did not significantly influence the eggshell thickness but modified the crystallographic structure of the effective layer height and palisade layer height.

The effects of Zn-Cu-Mn mixture on eggshell ultrastructure are due to their role in shell formation in the uterus. A large amount of calcium and carbonate ions in uterine fluid were transported from the blood to the uterine glands across epithelial cells, thereby continuously supplying the inorganic components required for eggshell mineralization. In addition, the better endometrial morphology such as higher mucosal folds height increased the ions transport and absorption and accelerated calcification (Ma et al., 2020). The conditions of endometrial affect the formation of the eggshell structure (Jonchere et al., 2012). We observed a dramatically increased mucosal fold and numerically increased epithelial height in uterus in the HC group when compared with the Sulphate group. The development and maintenance of the uterine mucosa were enhanced by dietary HC trace elements addition. As illustrated in the other studies, Zn improved the quality of epithelium in the oviduct because of its important role in protein synthesis (Tabatabaie et al., 2007). Furthermore, previous studies concluded that dietary mineral supplementations modulate eggshell structure by enhancing the GAG, uronic acid synthesis (Xiao et al., 2014), and the expression of glycoproteins (Zhang et al., 2017b) in the eggshell glands.

The hydroxychloride trace element mixture increased digestibility and availability and prevented from forming insoluble compound with other minerals and vitamins in the feed (Lu et al., 2010), thereby increased the absorption of Ca. Addition of hydroxychloride trace elements to the diet, the level of total calcium in serum increased, which probably accompanied with a higher concentration of calcium in uterine fluid that precipitated in forming of calcite thus facilitating eggshell mineralization (Hincke et al., 2012). The carbonic anhydrases (**CA**) are presented in cell membranes of tubular glands and capillary endothelium and its absence are associated with producing eggs with thinner shells (Wistedt et al., 2019; Abedini et al., 2017). Under the conditions of the present study, diet with minerals did not affect CA content in serum and CaBpD28k expression in shell gland of aging hens, which was partly consistent with the postulation that the CaBpD28k levels in eggshell gland was not affected by the mineral sources and levels (Li et al., 2018). Additionally, CA primarily takes part in the flow of ca^2+^ from the gut to the egg shell gland resulting in high HCO_3_ depositing Ca^2+^ in the egg shell gland (Yang et al., 2013). Furthermore, Tsai et al. (2016) found that trace element increased CA activity in serum, which was inconsistent with our finding. It seems that some of the contradictory results arose from minerals source and doses, hen breed and age, dietary composition and other factors (Min et al., 2018b).

Trace elements are essential to sustain production besides regulating antioxidant activities and immune systems (Rao et al., 2016). Availability of hydroxychloride minerals has been extensively studied in ruminants and poultry (Wagner, et al., 2016; Perez et al., 2017). It has been observed that synergistic interactions between hydroxychloride Mn and Cu increased SOD activity and contributed to antioxidant defense during an inflammatory challenge (Perez et al., 2017). Chicken SOD catalyzes the neutralization of superoxide radicals and serves to protect the cells against oxidative damage (Abreu and Cabelli, 2010).The positive influence of hydroxychloride Zn, Cu, and Mn mixture on ceruloplasmin in serum was observed in our study whereas SOD was not significantly affected, which was consistent with Yuan et al. (2011). Previous studies (Jarosz et al., 2018) confirmed the stimulating effect of copper in humoral response by increasing ceruloplasmin content. Furthermore, we found that in Sulphate and HC group, there was a statistical increase in *TNF-α* and a slight increase in *IL-22* content when compared with the CON group. Interestingly, almost all the preinflammatory cytokines in HC group were higher than those in the Sulphate group. The results were in agreement with Jarosz et al. (2018) who found that supplementation of feed with copper chelates activates mainly the Th1 cellular immune response and the response of peripheral blood T lymphocytes, as well as promotes secretion of cytokines, which are involved in potentiation and regulation of the immune response in birds. Wellinghausen et al. (1997) reported that trace element predominantly induced cytokines interleukin 1β (***IL-1β***), interleukin 6 (***IL-6***), and *TNF-α*, and therefore, had an immensely immune regulative capacity. The work of Elhamouly et al. (2018) found that the expression of proinflammatory cytokines *IL-1β, IL-6,* and *IFN-γ* was higher during the main phase of eggshell formation. The mechanism for this mainly was because that proinflammatory cytokines *IL-1β, IL-6* upregulated the expression of genes related to calcium and carbonate ion transport required for eggshell formation, but the content of CaBPD28K was reduced by cytokines in uterine tissue (Nii et al., 2018), which was consistent with our experiment. It is more generally believed that anti-inflammatory factors prevent host from being invaded by pathogens. Zn and Mn in hydroxychloride form synergistically enhanced *IL-1* expression in immune cells during an inflammatory response (Perez et al., 2017). But when it comes to aged hens, the situation is different. A previous study (Elhamouly et al., 2018) found that the higher expression of anti-inflammatory cytokines observed in aged hens may disrupt the balance of inflammatory cytokines. Therefore, it is possible that a well-controlled physiological inflammation occurs in the uterus during the process of eggshell formation when inorganic trace elements were added in the diet, especially the HC form of trace elements.

## CONCLUSIONS

In conclusion, hydroxychloride Zn, Cu, and Mn excelled sulphate in its beneficial effects for Haugh unit and albumen height from 70 to 73 wk as well as mucosal fold height and *TNF-α* expression. The hydroxychloride trace element Zn-Cu-Mn mixture modified eggshell ultrastructure partly by improving serum parameter, uterus morphology, and immune response. Further investigation should be conducted to study the role of trace elements in immune response of uterus and the relationship between minerals, uterus health, and eggshell quality.
